# Mass Spectrometry Imaging as a Potential Tool to Investigate Human Osteoarthritis at the Tissue Level

**DOI:** 10.3390/ijms21176414

**Published:** 2020-09-03

**Authors:** Yea-Rin Lee, Matthew T. Briggs, Mark R. Condina, Hamish Puddy, Paul H. Anderson, Peter Hoffmann, Julia S. Kuliwaba

**Affiliations:** 1 Clinical and Health Sciences, Health and Biomedical Innovation, University of South Australia, Adelaide, SA 5000, Australia; yea_rin.lee@mymail.unisa.edu.au (Y.-R.L.); paul.anderson@unisa.edu.au (P.H.A.); 2 Future Industries Institute, Mawson Lakes Campus, University of South Australia, Mawson Lakes, SA 5095, Australia; matthew.briggs@unisa.edu.au (M.T.B.); mark.condina@unisa.edu.au (M.R.C.); peter.hoffmann@unisa.edu.au (P.H.); 3 Discipline of Orthopaedics and Trauma, Adelaide Medical School, The University of Adelaide, Adelaide, SA 5000, Australia; 4 School of Medicine, Flinders University, Bedford Park, SA 5042, Australia; hamish.puddy@sa.gov.au

**Keywords:** osteoarthritis, proteomics, biomarkers, matrix-assisted laser desorption/ionisation mass spectrometry imaging

## Abstract

Osteoarthritis (OA) is the most common degenerative joint disease, predicted to increase in incidence year by year due to an ageing population. Due to the biological complexity of the disease, OA remains highly heterogeneous. Although much work has been undertaken in the past few years, underlying molecular mechanisms leading to joint tissue structural deterioration are not fully understood, with only few validated markers for disease diagnosis and progression being available. Discovery and quantitation of various OA-specific biomarkers is still largely focused on the bodily fluids which does not appear to be reliable and sensitive enough. However, with the advancement of spatial proteomic techniques, several novel peptides and proteins, as well as N-glycans, can be identified and localised in a reliable and sensitive manner. To summarise the important findings from OA biomarker studies, papers published between 2000 and 2020 were searched via Google Scholar and PubMed. Medical subject heading (MeSH) terms ‘osteoarthritis’, ‘biomarker’, ‘synovial fluid’, ‘serum’, ‘urine’, ’matrix-assisted laser desorption/ionisation’, ‘mass spectrometry imaging’, ‘proteomic’, ‘glycomic’, ‘cartilage’, ‘synovium’ AND ‘subchondral bone’ were selectively used. The literature search was restricted to full-text original research articles and written only in English. Two main areas were reviewed for OA biomarker studies: (1) an overview of disease-specific markers detected from different types of OA bio-samples, and (2) an up-to-date summary of the tissue-specific OA studies that have utilised matrix-assisted laser desorption/ionisation mass spectrometry imaging (MALDI-MSI). Overall, these OA biomarkers could provide clinicians with information for better the diagnosis, and prognosis of individual patients, and ultimately help facilitate the development of disease-modifying treatments.

## 1. Introduction

Osteoarthritis (OA) is one of the most common degenerative joint diseases that affected 303 million people globally in 2017 [1]. OA has a considerable impact on the individual patient, resulting in pain and disability, and has become a major health burden. Disease progression is associated with cartilage degradation, synovial inflammation and bone-related changes including osteophytes, subchondral sclerosis and bone marrow lesions. Disease rates typically increase year by year, due to an ageing population coupled with other well-known risk factors such as obesity, being female and joint injuries [2]. Despite its high prevalence and substantial public health impact, underlying molecular mechanisms remain incompletely understood. Understanding the biomolecular contribution and interaction between the joint tissues in the early stage of OA development is therefore crucial since biomolecular changes could be targeted to slow further progression of the irreversible structural deterioration. Theoretically, with the onset of OA, the metabolism of chondrocytes in cartilage and extracellular matrix (ECM) is disturbed, exceeding the production of matrix degradation enzymes [3]. The measurement of these reflects dynamic and quantitative changes in joint remodelling and OA disease progression. 

To date, OA treatments are predominantly restricted to symptomatic relief and inflammation management by analgesics and non-steroidal anti-inflammatory drugs, with joint replacement surgery as the only effective treatment option for late stage [4,5]. The diagnosis of OA relies heavily on the description of symptoms such as severities of pain, stiffness and joint space narrowing detected by X-ray [6,7]. However, plain X-ray provides limited information on non-mineralised tissues, such as articular cartilage, and does not allow the detection of biomolecular changes that presage cartilage, synovium and bone abnormalities. Given the limitations of currently available diagnostic tools for investigation of OA, it is not surprising that there has been considerable interest in the identification of disease-specific biomarkers that contribute to and interact with the joint tissue function. Establishing predictive biomarkers could therefore contribute to early detection of OA and the prediction of disease severity, which could further lead to the development of earlier treatment options.

There is a need for alternative imaging methods that can detect OA-disease biomarkers in a quantitative, reliable and sensitive manner. For this reason, recent research on OA biomarkers has moved to applying mass spectrometry imaging (MSI). MSI is an analytical technique used for determining the spatial distribution of biomolecules such as lipids, peptides, proteins, metabolites, glycans and other organic compounds in the tissue section while preserving tissue morphology [8]. Once mass spectra are acquired, it is possible to generate an ion intensity map that represents the spatial distribution and relative abundance of specific biomolecules, which can then be correlated to histological features. The main advantage of using MSI is the feasibility to study different tissue-types or regions without previous knowledge of the heterogeneity of the sample. MSI has already demonstrated its strengths, mostly in cancer-related research, for biomarker discovery and disease classification and drug monitoring. However, there is still a limitation of applying currently available MSI methods to cartilage-bone joint tissues due to the high degree of cross-linking between the ECM and collagen that blocks the access of particular enzymes such as trypsin and PNGase F [9]. The relevant proteins, peptides and post-translational modifications (PTMs), such as N-glycans, therefore, cannot be easily analysed and localised. 

Among the different MSI strategies, matrix assisted laser desorption/ionisation (MALDI)-MSI has recently demonstrated its ability to profile analytes on both fresh-frozen (FF) and formalin-fixed paraffin-embedded (FFPE) OA cartilage, bone and synovium tissues, which potentially opens new opportunities for the discovery of novel disease-specific analytes. These analytes could then be used to monitor or diagnose OA, thus enabling the translation from discovery to clinical application [10]. The focus of this review is on published peer-reviewed papers in the past 20 years relating to OA bodily fluid biomarkers, such as synovial fluid, serum and urine (between 2002 and 2020), and a narrative up-to-date summary of the OA tissue studies that utilise MALDI-MSI (between 2013 and 2020). With a better understanding of the literature, we could improve methodology to study specific OA biomarkers that are more compatible with the clinical and radiological findings.

## 2. OA Disease-Specific Biomarkers

Given the lack of effective diagnostic tools for early OA detection and evaluation of currently available treatments, there have been multiple studies carried out with the aim to discover novel biomarkers that could potentially be useful to monitor pathological developments in OA. These studies have been predominantly analyses of biological fluids, such as serum, urine and synovial fluid, as they are easily obtained from the body, and theorised to be rich sources of proteins [11].

Following an investigation of the literature, a list of potential OA biomarkers measured by either antibody-based immunoassay coupled with or without MS, two-dimensional (2D) electrophoresis or liquid chromatography (LC) has been summarised in Table 1. These different biomarkers can be identified from the cartilage, synovium and subchondral bone, and many of them are interchangeably associated with the metabolism of collagen and aggrecan in the cartilage [12,13]. So far, eight main biomarkers concerning Type II collagen metabolism have been identified. Six of them (i.e., C-telopeptide fragments of type II collagen; CTX-II, Type II collagen fragment; Helix-II, Cartilage type II collagen; C2C, Oxidative-related type II collagen; Coll2-1, Oxidative-related type II collagen in nitrated form; Coll2-1 NO2, Type II collagen neoepitope; TIINE) are for collagen degradation, and two of them (i.e., N-propeptide of collagen IIA; PIIANP, Carboxyl terminus propeptide of type II procollagen; PIICP) are for collagen synthesis. Among them, CTX-II seems to be the most promising clinical biomarker as it is degraded by cartilage-degrading enzymes as soon as OA occurs [14] and it is significantly correlated with the disease severity [15]. 

In addition, biomarkers related to a range of non-collagenous proteins have been identified that also play a role in other metabolic pathways in the joint; these include, glycoproteins, proteoglycans, metalloproteinases and advanced glycation end products [12]. Among them, a glycoprotein called cartilage oligomeric matrix protein (COMP) is the second most frequently studied OA biomarker and abundant in OA cartilage—but in other tissues (i.e., tendon, meniscus, ligament and synovial membrane) as well, found in both serum and synovial fluids [16]. The concentration of COMP is much higher in synovial fluids than in serum, indicating preferential release from the affected joints. COMP is the only biomarker that has been shown to be a statistically significant predictor of cartilage loss on knee magnetic resonance imaging (MRI) even after adjustment for age, gender and patient’s body mass index (BMI) [17]. In this study, the authors used data from a longitudinal study with 137 patients who experienced symptomatic knee OA. However, precisely what role it plays in OA pathogenesis remains unclear and requires further investigation. 

Further, OA is widely accepted as a disease of the whole joint affecting not only the cartilage but also the synovium and the subchondral bone. Changes in these structures are also found to be related to the disease, however, these tissues have received far less attention than the biomarkers from cartilage. Hyaluronan (HA) is a glycosaminoglycan formed by alternating units of glucosamine and glucuronic acid. It is a constituent of the synovial fluid and thought to contribute to the lubricating mechanisms. HA concentration can be measured in both synovial fluid and serum, and HA is the third most studied OA biomarker [16].

Finally, degradation of type I collagen during subchondral bone resorption can be reflected by elevated levels of N- and C-telopeptide fragments of collagen type-I (NTX-I and CTX-I). One study has found that both of these markers were significantly associated with reduced cartilage loss [18], and another study further correlated the significant association with progression of OA [19]. These results are still preliminary in terms of clinical use, and further characterisation is needed to determine their predictive capability.

## 3. Proteomic-Based Imaging Approaches in OA Joint Tissue Studies

The OA biomarkers discussed above are all proteins measured from the synovial fluid, serum, or urine. These markers have not been applied in general clinical practice yet, as there is only limited consensus about their predictive value [47]. Thus, different approaches are required to improve the specificity and validity of these markers. Advanced proteomics technology provides a powerful tool to allow us to build up a library of such factors that govern the cartilage-bone-synovium interplay. Bodily fluids have been frequently studied as mentioned before, whereas OA cartilage, bone and synovial tissues have been assessed in a handful of studies. 

**Figure 1:**
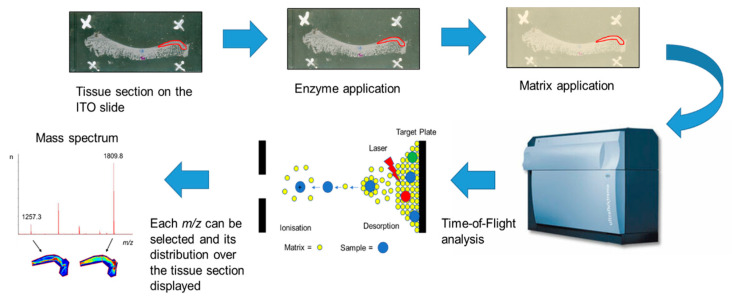
Schematic matrix-assisted laser desorption/ionisation mass spectrometry imaging (MALDI-MSI) workflow for fresh-frozen (FF) or formalin-fixed paraffin-embedded (FFPE) tissue using the “bottom-up” strategy [50]. Tissue is sectioned and mounted onto indium tin oxide (ITO) slides. FFPE tissue sections are deparaffinised followed by antigen retrieval. Then, an enzyme is sprayed directly onto the tissue section followed by matrix deposition, most commonly α-cyano-4-hydroxycinnamic acid (CHCA). Mass spectra are acquired across the whole tissue section or region of interest and the data are analysed using MSI software.

MALDI-MSI is the most versatile MSI platform that allows the in-situ detection of a large number of peptides, proteins and even PTMs. A general MALDI-MSI workflow is shown in Figure 1 [10,48,49]. In brief, either FF or FFPE tissue sections are mounted onto commercially available indium tin oxide (ITO) coated glass slides and processed accordingly. Then, slides are sprayed with a suitable enzyme for digestion and coated with matrix solution that assists in desorption and ionisation of the analytes. The tissue section is irradiated by a laser at discrete positions thereby generating a mass spectrum based on their *m/z* ratio for each measured position. From these data, MALDI-MSI allows the visualisation of hundreds to thousands of analytes localised to the tissue. Commonly, MALDI-MSI is used to spatially visualise analytes, which is then followed by liquid chromatography with tandem mass spectrometry (LC-MS/MS) characterisation of these analytes after extraction from consecutive tissue sections [48,49].

The application of MALDI-MSI to joint tissues is limited so far, due to the complex sample preparation and the lack of established protocols. Therefore, only a limited number of OA studies have been conducted using MALDI-MSI on articular cartilage, synovial membrane, and subchondral bone tissue, as demonstrated in Table 2.

### 3.1. MALDI-MSI Analysis of Articular Cartilage

Articular cartilage provides a direct insight into the pathogenesis of OA as it has been the most extensively investigated joint tissue in the past. Articular cartilage is mainly composed of ECM proteins, including collagens and proteoglycans, which may alter with disease state [56]. Robust MALDI-MSI work has identified various disease-specific markers that differentiate OA and healthy cartilage in humans [51], and markers that distinguish ageing (>15 years old) and young (4 years old) cartilage from OA cartilage in horses [52]. Compounds determined to be potential OA markers include COMP, haemoglobin-beta, phosphocholine and fibronectin, among other proteins. These findings were verified by immunohistochemistry (IHC). Interestingly, these disease-specific proteins were predominantly found in the deep layer of cartilage, which is close to the subchondral bone, however, further validation in larger cohorts of human samples with the same workflows is required.

### 3.2. MALDI-MSI Analysis of Synovial Membrane

Relatively less attention has been directed at the synovial membrane in OA studies given the easier sampling of synovial fluid and the assumed greater importance of cartilage in OA pathology. In an earlier study, Kriegsmann et al. demonstrated the presence of calgranulins, defensins and thymosins, which were detected from various synovial compartments, including the synovial lining and sub-lining layer, from rheumatoid arthritis (RA) tissues, but not from OA tissues [53]. Interestingly, higher expression of thymosins responsible for T-lymphocyte maturation, suggested the involvement of lymphoid cells in the synovial pathology of RA, but not so much in OA. Shortly after, Cillero-Pastor et al. sought to characterise the proteome of OA synovial membrane using MALDI-MSI [54]. While recognising that the experimental protocol limited the detection of most proteins, specifically those of low abundance, the study concluded there was differential expression of haemoglobin subunits, biglycan, fibronectin and actin, which were further validated by IHC [54]. At present, studies focused on the synovium are limited, and a greater understanding of the OA proteome and inflammatory processes within and between tissues is necessary. 

### 3.3. MALDI-MSI Analysis of Subchondral Bone

It is well established that the homeostasis of articular cartilage relies on the biochemical and biomechanical interplay with the underlying subchondral bone [57]. It was proposed several decades ago that hardening of subchondral bone would increase the “wear and tear” risk of articular cartilage. Yet the factors or mediators that govern the hypertrophic changes of subchondral bone in OA remain largely unknown. The importance of subchondral bone in OA has only recently become better understood. As the interplay between all joint tissues is elucidated, there is emerging evidence that subchondral bone may exhibit changes early in the disease process [58]. There are technical challenges facing subchondral bone proteomics and development of a reliable workflow. Mareddy et al. sought to create a reference map of bone marrow mesenchymal stem cells in non-diseased tissue utilising a combination of MALDI-MSI and western blot for validation [59]. This study demonstrated the application potential of MALDI-MSI in analysing bone tissue. 

In a recent study conducted by Briggs et al., several challenges have been overcome with regard to hard-fragile bone tissue. N-glycans, which are protein PTMs, were identified from FFPE OA cartilage-bone tissue using a combination of MALDI-MSI and liquid chromatography/electrospray ionisation tandem mass spectrometry (LC-ESI-MS/MS) [55]. This complimentary approach was first applied to N-glycans using FFPE OA cartilage–bone tissue sections. The analysis of N-glycans is especially relevant in OA since these analytes are associated with the progression of the disease [60]. This study identified about 40 N-glycans from OA cartilage and its underlying bone and bone marrow by MALDI-MSI [55]. Among them, oligomannose N-glycans showed differential spatial distribution between the OA tissues. Particularly, (Man)_3_ + (Man)_3_ (GlcNAc)_2_ was found to be increased in cartilage compared to subchondral bone [55]. In addition, OA patients with different bone marrow lesion stages were discriminated according to the sialylated N-glycan, (NeuAc)_2_ (Hex)_2_ (HexNAc)_2_ + (Man)_3_ (GlcNAc)_2_ [55]. These results provide evidence of a potential cartilage degradation marker. However, the sample preparation protocol still requires further development.

Overall, these studies demonstrate that MALDI-MSI is a novel technology to investigate joint tissues and potential peptide or N-glycan markers of OA disease progression. However, protein identification and extraction from the MALDI-MSI workflow is still a challenge as the properties of joint tissues contain a low cell density and a high abundance of large molecular weight proteins and collagens in the ECM. Therefore, different approaches are needed to improve methodologies. 

## 4. Challenges and Future Directions for Biomarkers in OA Joint-Derived Samples

The discovery of potential OA disease-specific biomarkers in the past has been limited to mainly focusing on either cartilage tissue or bodily fluids, as these are relatively less complicated to collect and thus a cost-effective way to study. Collagens, predominantly type II, and proteoglycans, often overwhelm the intensity levels of other proteins within the articular cartilage. Other anionic macromolecules such as aggrecan and HA, and cellular proteins from chondrocytes, also hinder the identification of lower abundant ECM proteins. This challenging ECM protein network makes the extraction and analysis of OA-affected joint tissues technically difficult to achieve. In addition, the collection of bodily fluid samples in most past studies was not standardised and markers were often not considered for demographic variables such as age, gender and patient’s BMI.

The appropriate methodologies to identify reliable OA biomarkers need to be continuously optimised to better understand the complex pathogenesis of OA. A recent study by Angel et al. demonstrated a novel MALDI-MSI breakthrough where collagenase is used instead of trypsin to digest ECM proteins [61]. This allowed spatial visualisation of collagen and elastin peptides in the tumour microenvironment of FFPE cancer tissues. This novel workflow would be useful to apply to OA articular cartilage and subchondral bone tissue samples. Despite all of these limitations, the main advantage of “bottom-up” MALDI-MSI strategies is the ability to spatially map hundreds of analytes across FF or FFPE tissue without the use of labelling and staining techniques. Recently improved high-throughput technologies will potentially offer the possibility to characterise the entire complexity of the OA-affected joint tissue proteome, glycome or metabolome which could contribute to a better understanding of OA disease progression and development of future disease-modifying treatments [62].

There is intense interest in multi-modal imaging analysis of proteins and N-glycans to understand the protein populations undergoing specific glycosylation changes in single FFPE tissue sections [63]. Recent studies from Peggi et al. and Heijs et al. also describe the process of preparing the same FFPE tissue section for imaging of N-glycans followed by proteolytic peptides [61,64], which opens a door to the discovery of multiple novel disease-specific markers. Thus, future research should focus on analysis of N-glycans and glycoproteins from OA-affected joint tissues as they could provide “molecular signatures”. It is well-established that glycoproteins regulate ECM protein turnover and hinder the metabolism of chondrocytes, which increases their relevance to OA progression. To the best of our knowledge, this multi-modal approach has never been conducted in OA joint-derived tissues and this should be the next generation tool for identifying differentially present N-glycans or ECM glycoproteins within a single tissue section. An optimal combination of MSI techniques with further development of different methodologies may offer new opportunities to tackle these challenges and provide a better insight into the molecular mechanisms that lead to OA and other rheumatic pathologies.

## 5. Conclusions

OA is a disease of complex aetiology resulting in organ failure of the entire joint structure. Degeneration of articular cartilage, synovial immunopathogenesis and structural changes in subchondral bone are key factors in the mechanisms leading to joint damage in OA. COMP, aggrecan, matrix metalloproteinases, collagens and complement factors, in particular, have been reported as potential biomarkers for OA joint tissue changes. However, these biomarkers have been discovered predominantly from bodily fluids and have limited value in diagnosis and prognosis or in monitoring effect of treatment. Therefore, further investigation aimed at characterising the proteome, glycome and metabolome at the tissue level and correlations with adjacent tissues within the OA joint are highly recommended and the most promising approaches to discovery of novel OA biomarkers. Recently, there is a great deal of interest in MSI-based approaches to identify and localise OA biomarkers within tissue sections, and with significant advances in this field, the challenges of hard-complex joint tissues could be resolved. Discovering and validating potential OA biomarkers using well-characterised patient cohorts could lead to assisting clinicians to identify different OA patient phenotypes and develop comprehensive non-invasive methods to evaluate OA patients.

## Figures and Tables

**Table 1:** Potential biomarkers currently being investigated for the evaluation of osteoarthritis (OA) and presumed pathological process, measured from synovial fluid, serum or urine.

OA-Affected Tissue	Sample Type	Molecule Type	Biomarker(s)	References
Articular Cartilage	Synovial Fluid	Type II collagen	C-propeptide of collagen type II (PIICP)	Sugiyama et al., 2003 [20]
	Serum		N-propeptide IIA of type II collagen (PIIANP)	Kraus et al., 2017 [21]
	Urine, Synovial Fluid		C-terminal telopeptide of collagen type II (CTX-II)	Rotterud et al., 2014 [22]; Sowers et al., 2009 [23]; Lohmander et al., 2003 [24]
	Serum, Synovial Fluid, Urine		Type II collagen cleavage product (C2C)	Kumahashi et al., 2015 [25]; He et al., 2014 [26]
	Serum		Matrix metalloproteinase-derived fragment of type II collagen (CIIM)	Bay-Jensen et al., 2011 [27]
	Urine		Helical peptide of type II collagen (HELIX-II)	Charni et al., 2005 [28]
	Serum	Type X collagen	C-terminus of collagen type X (C-Col-10)	He et al., 2014 [29]
	Serum	Aggrecan	Aggrecan chondroitin sulfate epitope 846	Ma et al., 2018 [30]
	Synovial Fluid		Aggrecanase-generated aggrecan fragment with the ARGS neoepitope	Germaschewski et al., 2014 [31]
	Serum	Non-collagenous and aggrecan proteins	Cartilage oligomeric matrix protein (COMP and its deaminated form D-COMP)	Ma et al., 2018 [30]; Verma et al., 2013 [32]; Sowers et al., 2009 [23]
	Serum, Synovial Fluid		Pentosidine	Senolt et al., 2005 [33]
	Serum		Follistatin-like protein (FSTL-1)	Wang et al., 2011 [34]
	Serum		Fibulin (peptides of fibulin 3, Fib3-1, -2)	Henrotin et al., 2012 [35]
	Serum	Proteolytic enzymes	Matrix metalloproteinases (MMP-3, -9)	Li et al., 2012 [36]
	Synovial Fluid		Matrix metalloproteinases (MMP-1, -13)	Ozler et al., 2016 [37]; Rubenhagen et al., 2012 [38]
	Serum		A disintegrin and metalloproteinase with thrombospondin-like motif 4 (ADAMTS-4)	Li et al., 2014 [39]
	Synovial Fluid	Proteolytic enzyme inhibitors	Tissue inhibitor of matrix metalloproteinase (TIMP-1)	Hegemann et al., 2003 [40]
Synovium	Serum	Non-collagenous proteins	Hyaluronan (Hyaluronic acid; HA)	Sasaki et al., 2013 [41]
	Synovial Fluid		Cartilage glycoprotein 39 (YKL-40)	Guan et al., 2015 [42]
	Urine	Type III collagen	Glucosyl-galactosyl pyridinoline (Glc-Gal-PYD)	Jordan et al., 2006 [43]
Subchondral Bone	Serum	Type I collagen	Aminoterminal propeptide of collagen type I (PINP)	Kumm et al., 2013 [44]
	Serum, Urine		C-telopeptide fragment of collagen type-I (CTX-I)	Nikahval et al., 2016 [45]; Bettica et al., 2002 [19]
	Urine		N-telopeptide fragment of collagen type-I (NTX-I)	Bettica et al., 2002 [19]
	Urine		Non-isomerised C-telopeptide fragment of collagen type-I (Alpha-CTX-I); Isomerised C-telopeptide fragment of collagen type-I (Beta-CTX-I)	Kraus et al., 2017 [21]
	Urine		Pyridinoline (PYD)	Ok et al., 2018 [46]
	Urine		Deoxypyridinoline (DPD)	Ok et al., 2018 [46]
	Serum	Non-collagenous protein	Osteocalcin (OC)	Kumm et al., 2013 [44]
	Urine		Mid-fragments of osteocalcin (Mid OC)	Kumm et al., 2013 [44]

**Table 2:** A summary of OA-derived tissue studies using matrix-assisted laser desorption/ionisation mass spectrometry imaging (MALDI-MSI).

OA-Affected Tissue	Origin	Disease	Age in Years	Specific Marker(s) Identified	Validation	Reference
Articular Cartilage	Human	OA (*n* = 10) vs. HC (*n* = 10)	OA (51–84 years old), HC (51–91 years old)	Aggrecan core protein (ACAN), Biglycan (BGN), Cartilage intermediate layer protein 1 (CILP-1), Cartilage oligomeric matrix protein (COMP), Collagen alpha-1(II) chain (COL-2A-1), Decorin (DCN), Fibromodulin (FMOD), Fibronectin (FN), Prolargin, Protein embryonic large molecule derived from yolk sac (ELY-S)	IHC	Cillero-Pastor et al., 2013 [51]
	Horse	OA (*n* = 3) vs. Young (*n* = 3) and Old (*n* = 3)	OA (greater than 15 years old; 52 years in human), Young (4 years old; 14 years in-human), Old (greater-than 15 years old; 52 years in human)	Biglycan (BGN), Cartilage intermediate-layer protein-1 (CILP-1), Cartilage oligomeric-matrix protein (COMP), Collagen alpha-1(II)-chain (COL-2A-1), Collectin-43 (CL-43), Chondroadherin, Fibronectin (FN), Matrilin-3 (MATN-3), Melanoma inhibitory activity-3 (MIA-3)	IHC	Peffers et al., 2014 [52]
Synovial Membrane	Human	OA (*n* = 3) vs. RA (*n* = 3)	NA	Calgranulins, Defensins, Thymosins	NA	Kriegsmann et al., 2012 [53]
	Human	OA (*n* = 3) vs. HC (*n* = 3)	OA (69–82 years old), HC (60–78 years old)	Actin aortic smooth muscle (ACTA), Biglycan (BGN), Fibronectin (FN), Haemoglobin subunit-alpha-2 (HBA-2), Haemoglobin subunit beta (HBB)	IHC	Cillero-Pastor et al., 2015 [54]
Subchondral Bone	Human	OA with bone marrow lesions (*n* = 2) vs. OA without bone marrow lesions (*n* = 1)	One male aged 52 years, two females aged 68 and 74 years	N-glycans	LC-ESI MS/MS	Briggs et al., 2016 [55]

Abbreviations: Osteoarthritis, OA; healthy control, HC; immunohistochemistry, IHC; not applicable, NA; liquid chromatography/electrospray ionisation tandem mass spectrometry, LC-ESI MS/MS.

## Abbreviations

OA	osteoarthritis
ECM	extracellular matrix
MSI	mass spectrometry imaging
PTMs	post-translational modifications
MALDI-MSI	matrix-assisted laser desorption/ionisation mass spectrometry imaging
FF	fresh-frozen
FFPE	formalin-fixed paraffin-embedded
2D	two-dimensional
LC	liquid chromatography
CTX-II	C-telopeptide fragments of type II collagen
Helix-II	type II collagen fragment
C2C	cartilage type II collagen
Coll2-1	oxidative-related type II collagen
Coll2-1 NO2	oxidative-related type II collagen in nitrated form
TIINE	type II collagen neoepitope
PIIANP	N-propeptide of collagen IIA
PIICP	carboxyl terminus propeptide of type II procollagen
COMP	cartilage oligomeric matrix protein
MRI	magnetic resonance imaging
BMI	body mass index
HA	hyaluronan
NTX-I	N-telopeptide fragments of collagen type-I
CTX-I	C-telopeptide fragments of collagen type-I
ITO	indium tin oxide
LC-MS/MS	liquid chromatography with tandem mass spectrometry
IHC	immunohistochemistry
ITO	indium tin oxide
RA	rheumatoid arthritis
LC-ESI-MS/MS	liquid chromatography/electrospray ionisation tandem mass spectrometry
